# *Vatica najibiana* (Dipterocarpaceae), a new species from limestone in Peninsular Malaysia

**DOI:** 10.3897/phytokeys.98.23903

**Published:** 2018-05-10

**Authors:** Abdul Rahman Ummul-Nazrah, Mohd Amin Mohd Hairul, Imin Kamin, Ruth Kiew, Poh Teck Ong

**Affiliations:** 1 Forest Research Institute Malaysia, 52109 Kepong, Selangor, Malaysia

**Keywords:** Dipterocarpaceae, *Vatica*, Kelantan, Pahang, limestone hills, oil palm, logging

## Abstract

*Vatica najibiana* Ummul-Nazrah (Dipterocarpaceae), from the Relai Forest Reserve, Gua Musang, Kelantan and Gua Tanggang, Merapoh, Pahang, is described and illustrated. This species is Endangered and known from small populations restricted to two isolated karst limestone hills. The type locality, Relai Forest Reserve limestone, is currently under threat from encroaching oil palm plantations and ongoing logging, which, if it continues, will threaten the Kelantan population with extinction. The morphology of *V. najibiana* and the similar V. odorata subsp. odorata and *V. harmandiana* is compared.

## Introduction

In Peninsular Malaysia, *Vatica* L., known in Malay as *resak*, includes 32 species (Saw 2002; Ashton and Appanah 2004; El-Taguri and Latiff 2010, 2012; Tan et al. 2014; Chua et al. 2015). It is a genus of understorey and main canopy trees from lowland forest to hill dipterocarp forest but it also occurs in coastal peat swamp and other swampy areas with only *V. harmandiana* Pierre and *V. kanthanensis* Saw restricted to limestone habitats. In Ashton and Appanah (2004) and Chua et al. (2015), *V. harmandiana* was called *V. cinerea* King that Pooma et al. (2017, p. 670) argued was a synonym of *V. harmandiana*. The genus *Vatica* is distinctly different from other Malayan genera of Dipterocarpaceae. Most *Vatica* species are small or medium-sized trees, unbuttressed with smooth bark and leaves with reticulate tertiary venation. There are two sections in *Vatica*: sect. Vatica with equal fruit calyx lobes and sect. Sunaptea with unequal fruit calyx lobes. This new Vatica species belongs to sect. Sunaptea, which now includes ten species.

This new *Vatica* species was discovered on the summit of a karst limestone hill within the Relai Forest Reserve, Gua Musang District, Kelantan, during the biodiversity survey of the flora of five sizeable limestone hills scattered within the FELDA Chiku oil palm plantation (Kiew et al. in prep.). The impetus for the survey was the issuing of a licence to quarry the largest hills named FELDA Chiku 7 and FELDA Chiku 8 to supply limestone to a new cement factory reputed to be the largest in SE Asia (Utusan Online 2015). The aim of the survey was to document the flora of the two hills scheduled for quarrying, which were previously hardly known botanically and to test the assertion by the cement company that protecting another nearby hill, FELDA Chiku 4, would compensate for biodiversity lost from the two larger hills. None of the limestone hills within the FELDA Chiku plantation is legally protected and all are currently under threat from disturbance associated with the oil palm plantation (clearing and burning the limestone forest around the base of the hills, grazing by free ranging cattle, hunting of the protected serow, *Capricornis sumatraensis*, collecting orchids etc.). Due to these threats, one species, *Impatiens chikuensis*, that is a strict endemic known only from FELDA Chiku 5 and 8, faces extinction (Kiew 2016).

The limestone hill flora in Peninsular Malaysia is being exploited and disturbed by quarrying, clearing the surrounding forest for agricultural plantations or burning limestone vegetation during land clearing, disturbance associated with caves, the establishment of temples and resorts, as well as from recreational and tourism activities (Kiew 1997). Kiew et al. 2017 demonstrated that no single hill has more than a fraction of limestone flora and that 192 species are endangered being known from less than five localities limestone hills, for example *Monophyllaea musangensis* A. Weber (Weber 1998), *Gymnostachyum kanthanense* Kiew, *Meiogyne kanthanensis* Ummul-Nazrah & J.P.C.Tan and *Vatica kanthanensis* Saw (Tan et al. 2014), *Impatiens glaricola* Kiew and *Impatiens vinosa* Kiew (Kiew 2016). Therefore, limestone hills are one of the most threatened vegetation types in Peninsular Malaysia and are recognised nationally as Environmentally Sensitive Areas because of their high biodiversity and vulnerability (73 of the 445 hills are the sites of active or former quarries, Liew et al. 2016). In addition, many karsts are still incompletely known botanically meaning that new species await discovery. For example, during the botanical survey of the Chiku limestone (Kiew et al. in prep.), several rare and endangered species were discovered, including this new species.

In determining the identity of the specimens, we discovered that the species had in fact already been collected from Gua Tanggang (a.k.a. Tagang) in Merapoh, Pahang, a limestone hill about 40 km south of the Relai Forest Reserve limestone but that it had been incorrectly identified as *Vatica cinerea* King (now *V. harmandiana*), a species restricted to NW Malaysia (Chua et al. 2010).

## Materials and methods

The new *Vatica* species was discovered on a limestone hill (5.024478 N, 102.114360 E, Ktn 50, numbering follows Price 2014) in the Relai Forest Reserve (Ktn 50), Gua Musang District, Kelantan. Herbarium specimens were collected and a photographic record was made. The population was in mature fruit but a few old dried flowers were obtained. The extensive collection of Dipterocarpaceae in the Kepong Herbarium (KEP) was used for comparison and for measurements of similar species and the other specimen of this new taxon (FRI 44774), previously collected from Gua Tanggang (a.k.a. Tagang), Merapoh, Pahang was examined in detail. The description of the new species was compared with similar species in standard texts (Saw 2002; Ashton and Appanah 2004; El-Taguri and Latiff 2010, 2012; Tan et al. 2014; Chua et al. 2015). The description is based on field observations and comparison by using KEP herbarium specimens. The provisional conservation assessment is based on the IUCN Red List Categories and Criteria Version 3.1 (IUCN 2012).

## Taxonomy

### Vatica najibiana

Taxon classificationPlantaeMalvalesDipterocarpaceae

Ummul-Nazrah sp. nov.

urn:lsid:ipni.org:names:77178683-1

Figures 1 , 2

#### Diagnosis.

Amongst the Vaticas with a half inferior ovary, it groups with *Vatica harmandiana* and V. odorata (Griff.) Symington subsp. odorata. *Vatica harmandiana* occurs on limestone hills and rocks but is different in having leaves that are elliptic-lanceolate, leaf base cuneate and nut diameter 7–10 mm as oppose to the obovate-elliptic leaf, leaf base cordate-subcordate and nut diameter of 5–6 mm in *V. najibiana*. Vatica odorata subsp. odorata is closely similar to the new species but can be separated by its elliptic-oblong leaf, leaf base obtuse, leaf apex acuminate, nut diameter 8–9 mm and occurrence in lowland and hill forest (Table 1).

**Table 1.** Differences between *Vatica najibiana*, V. odorata subsp. odorata and *V. harmandiana*.

Character	*V. najibiana*	V. odorata subsp. odorata	*V. harmandiana*
Habit	Small tree to 5–7 m	Tall tree to 24 m	Tree, 15–24 m
Leaves			
Petiole indumentum	Dark brown	Mid brown (reddish-brown)	Pale brown
Lamina shape	Obovate to elliptic	Elliptic to oblong	Elliptic to lanceolate
Lamina size (cm)	(3–)5–10.2 × 1.5–5	8–16 × 2.7–6	5.2–12 × (1.8–)2–5
Lamina base	Cordate to subcordate	Obtuse	Cuneate
Lamina margin	Recurved	Not recurved	Not recurved
Lamina apex	Acute	Acuminate	Blunt to acute
No. of lateral veins (pairs)	(6–)7–10	9–15	7–8
Fruits			
Calyx lobes length (cm)	2.3–3.3 × 0.5–0.8	4–5.5 × 1–1.5	2.6–7 × 1–1.8
Nut Diameter (mm)	5–6	8–9	7–10
Habitat	Limestone only	Lowland and hill forest	Limestone only

#### Type.

Peninsular Malaysia. Kelantan, Gua Musang District, Relai Forest Reserve (Ktn 50), 05°02'47.8"N, 102°11'43.6"E, 19 October 2016, Ummul-Nazrah et al. FRI 86369 (holotype KEP!; isotypes K!, SAN!, SING!).

**Figure 1.**
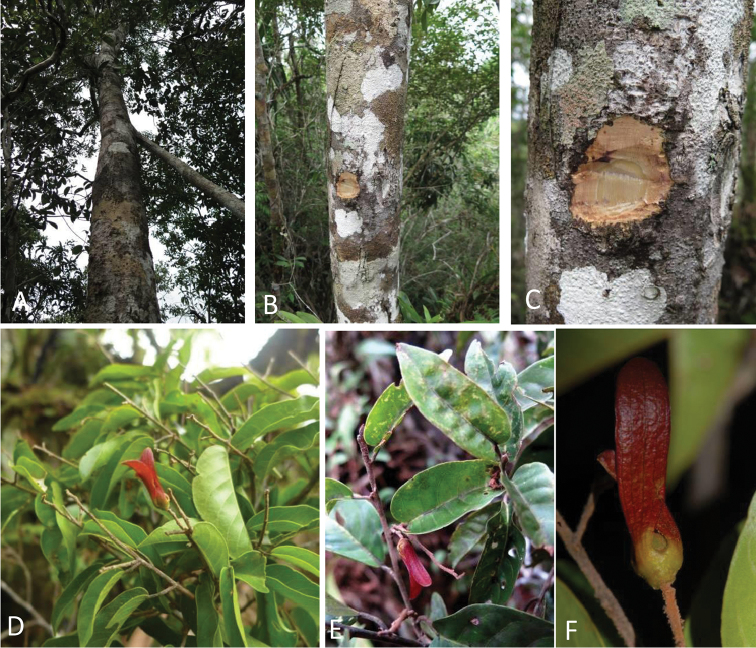
*Vatica najibiana*. **A** Plant in its natural habitat **B** Bole **C** Inner bark **D–E** Leafy shoots with infructescences **F** Fruit. (Photographs by K. Imin & A.R. Ummul-Nazrah).

**Figure 2.**
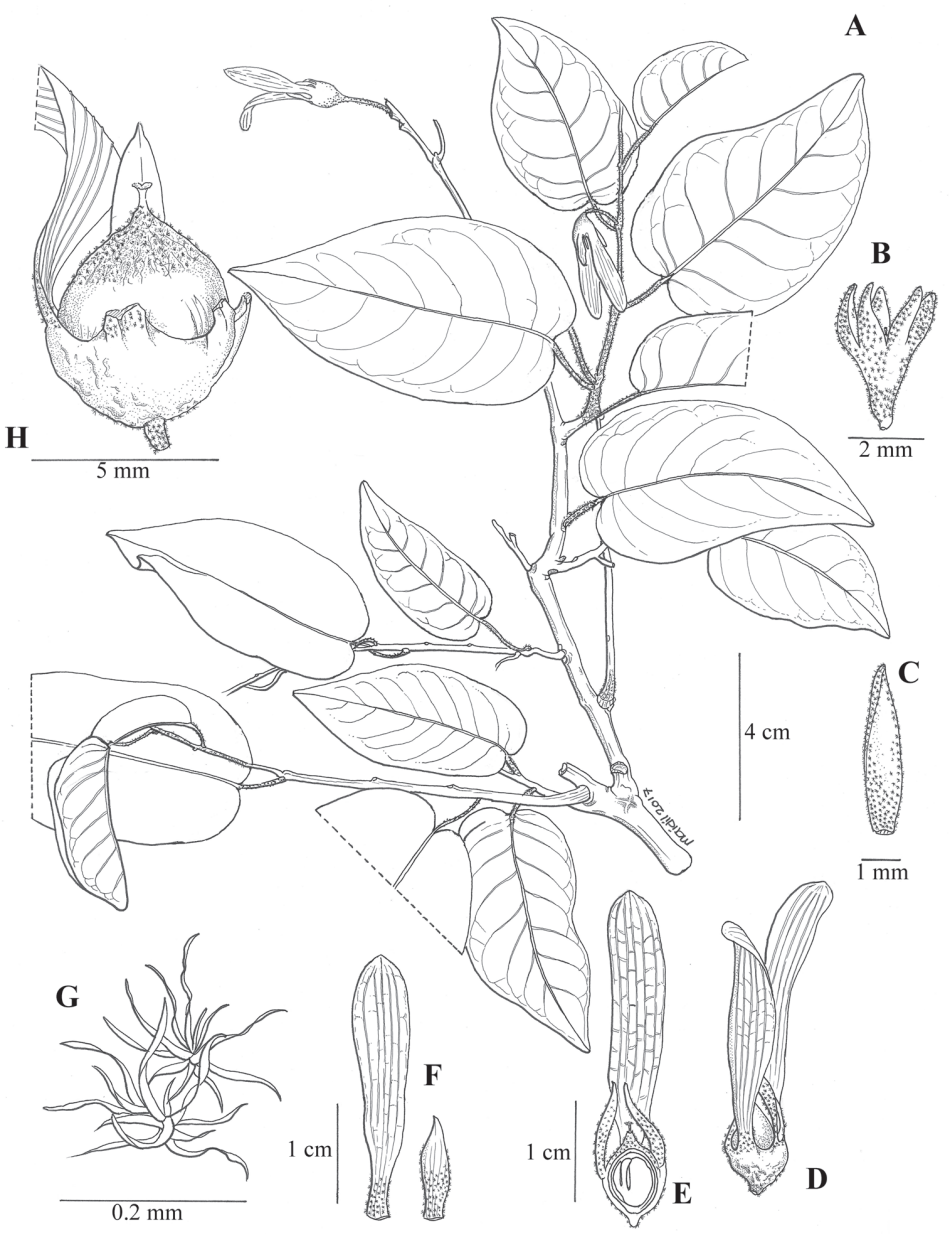
*Vatica najibiana*. **A** Leafy shoot with fruits **B** Calyx **C** Petal **D–E** Fruit **F** Long & short calyx lobes of fruit **G** Stellate hair **H** Fruit nut. (Drawn by N. Mohamad-Aidil from Ummul-Nazrah et al. FRI 86369).

#### Description.

Small tree, 5–7 m tall; bole to 15–17 cm diameter, without buttresses. **Bark** smooth with faint horizontal rings, dark brown with white lichen patches; inner bark pale yellow, exuding clear sap when cut. **Twigs** robust, 3–5 mm diameter, covered with 6–15-armed stellate hairs, 94–169 µm diameter, glabrous when mature, older twigs terete. **Leaves** when young brown rusty beneath, glabrous when mature; petioles 0.8–1.5 cm long, 0.1–0.2 cm wide, densely covered with stellate hairs, caducous when mature, drying dark brown; lamina obovate to elliptic, (3–)5–10.2 × 1.5–5 cm, thickly chartaceous, bullate, green on both surfaces when fresh, base cordate to subcordate, margin entire and recurved, apex acute; midrib prominent on both surfaces; lateral veins (6–)7–10 pairs, prominent below, slightly raised and visible above, ascending to margin; intercostal veins reticulate-scalariform and slightly conspicuous on both surfaces. **Flowers**: (dry) pedicels with velvety brown stellate hairs; calyx 5-lobed, elliptic, 4–7 × ca. 1 mm, densely covered with stellate hairs on both surfaces, apex acute; petals narrowly elliptic, ca. 6 × 2 mm, glabrous outside, inside from base to tip completely covered with 6–10-armed stellate hairs, 77–120 µm diameter. **Infructescence** axillary, near apex of leafy shoot, ca. 4 cm long, densely covered with rusty stellate hairs, branching once or twice, densely covered with stellate hairs; first branches with 1–7 fruits along axis, nodes 4–5 mm apart. **Fruits**: stalks 1–2 mm long, ca. 1 mm thick at base, covered with caducous stellate hairs; in life mature calyx red-brown, chartaceous, lobes 5, 2–3 larger than rest, attached to half inferior ovary, above forming a cup, glabrous outside, inner part at base completely covered with stellate hairs, lobes elliptic, apex rounded with 5 longitudinal prominent veins on the adaxial surface, 2.3–3.3 × 0.5–0.8 cm; shorter lobes 0.8–1 × ca. 0.2 cm; nut ovoid, 5–6 mm diameter, with persistent stigma, densely covered with stellate hairs, half hidden within calyx.

#### Distribution.

Endemic in Peninsular Malaysia, known only from Kelantan (Relai Forest Reserve, Gua Musang) and Pahang (Gua Tanggang, Merapoh).

#### Etymology.

This species is named in honour of the Prime Minister of Malaysia, Dato’ Sri Mohd Najib bin Tun Abdul Razak, for his strong interest in nature conservation and protection of the environment.

#### Provisional conservation status.

**Figure 3.**
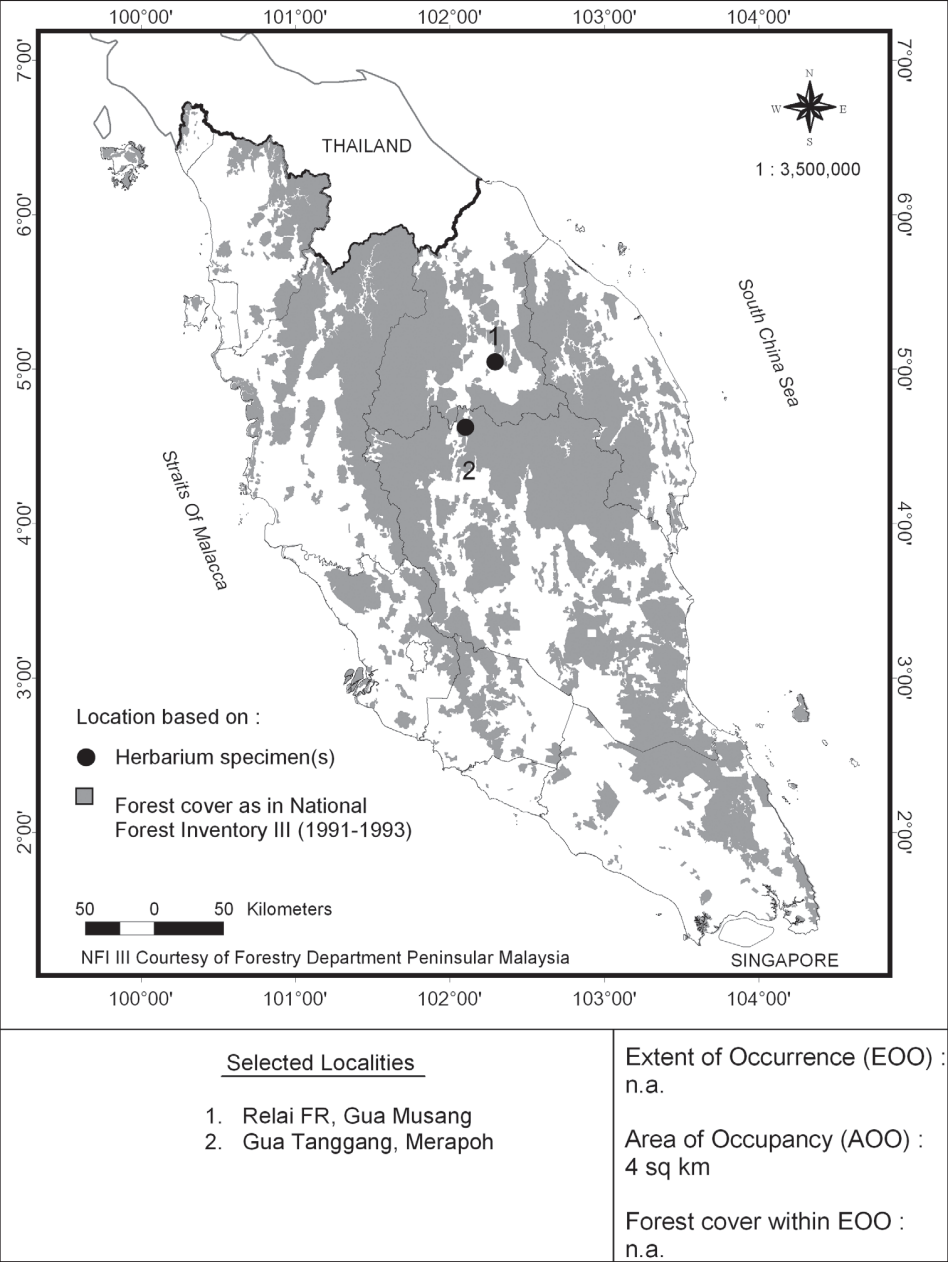
Distribution of *Vatica najibiana* in Peninsular Malaysia.

Endangered B2ab(iii). This species is known from the summit of two isolated karst limestone hills in Relai Forest Reserve, Gua Musang District, Kelantan and Gua Tanggang, Merapoh, Pahang, about 40 km apart (Liew et al. 2016). Together they have an area of occupancy of less than 10 km^2^ (Figure 3). The Relai Forest Reserve is classified as a permanent forest reserve but is currently threatened by encroachment by oil palm plantations that pose a high risk of burning to the limestone vegetation, as well as disturbance from ongoing logging in the Sungai Relai Forest Reserve. Gua Tanggang in Merapoh, on the other hand, is situated outside of Taman Negara which means that it is not in a protected area.

#### Habitat.

It is an emergent tree on the rugged summit of karst limestone at 178–520 m altitude growing in rock fissures with a thick layer of leaf litter.

#### Phenology.

Fruiting specimens were collected in Relai Forest Reserve in October and Gua Tanggang in early August; complete flowers not seen but in October, many calyces and a few petals were collected.

#### Additional specimen examined.

Peninsular Malaysia. Pahang, Lipis District, Merapoh, Gua Tanggang, 4.410000N, 102.055000E, 520 m alt., 6 August 1996, Saw et al. FRI 44774 (KEP!)

## Supplementary Material

XML Treatment for Vatica najibiana
